# SG-APSIC1112: The quality enhancement in sterilization processes at Naresuan University Hospital

**DOI:** 10.1017/ash.2023.105

**Published:** 2023-03-16

**Authors:** Kanokporn Wilachai

**Affiliations:** Central Sterilizing Services Association, Naresuan University Hospital, Tha Pho, Phitsanulok, Thailand

## Abstract

**Background:** Disinfection and sterilization of medical devices, instruments, and medical supplies are a crucial part of hospital infection control. The significant problems include insufficient cleaning, incorrect registration in the request form, no basic cleaning at the point of use (POS), and incorrect labelling. These problems were analyzed according using the fishbone diagram process. **Objectives:** We sought to decrease insufficient cleanliness of items after washing, to increase data accuracy in transferring contaminated devices from wards (from end users), to increase the rate of decontamination at the point of use, and to decrease incorrect labelling on instrument packaging. **Method:** The Community of Practice in Sterilization (CoP Sterile) team revised the disinfection and sterilization system of the Naresuan University Hospital Central Sterile Supply Department (CSSD) using root-cause analysis of subprocess problems and implementing prioritized solutions. A regular monthly meeting was set up to ensure active response, and closed monitoring was performed to ensure the implementation of the revised protocol according to the plan–do–study–act (PDSA) process. **Results:** From 2019 to 2021, the percentages of annual insufficient cleanliness of items after washing decreased from 4.8% to 3.6% to 3.1% each year. The percentages of incorrect request forms decreased from 14.59% to 2.91% to 1.84% during these same years. The percentages of decontamination ignorance at the point of use decreased from 3.13% to 0.12% to 0.03% from 2019 to 2021. The percentages of incorrect labelling were 0.013%, 0.008%, and 0.013% each year. **Conclusions:** The CoP Sterile team used quality improvement tools and regular monitoring to achieve reductions in insufficient cleanliness and incorrect request forms and to increase knowledge of decontamination procedures.

